# Progress and experiences of implementing an integrated disease surveillance and response system in Somalia; 2016–2023

**DOI:** 10.3389/fpubh.2023.1204165

**Published:** 2023-09-07

**Authors:** Steven Ssendagire, Mary Joan Karanja, Abdulkhadir Abdi, Mutaawe Lubogo, Abdullah Azad Al, Khadija Mzava, Abdinasir Yusuf Osman, Ahmed Mohamed Abdikarim, Mohamed Abdirahman Abdi, Abdirahman Mohamed Abdullahi, Abdirizak Mohamed, Hussein Sheik Ahmed, Nuralein Yusuf Hassan, Aden Hussein, Aisha Daud Ibrahim, Abdullahi Yusuf Mohamed, Ibrahim Mohamed Nur, Mukhtar Bulale Muhamed, Mohamed Abdelrahman Mohamed, Fawziya Abikar Nur, Hassan Sheik Ahmed Mohamed, Mohamed Mohamud Derow, Abdifatah Ahmed Diriye, Sk Md Mamunur Rahman Malik

**Affiliations:** ^1^World Health Organization Country Office, Mogadishu, Somalia; ^2^Health Information Strengthening Project, Dar es Salaam, Tanzania; ^3^Federal Ministry of Health, Mogadishu, Somalia; ^4^The Royal Veterinary College, University of London, Hatfield, United Kingdom; ^5^National Institute of Health, Mogadishu, Somalia

**Keywords:** integrated disease surveillance and response, implementation progress, lessons, next steps, Somalia

## Abstract

**Introduction:**

In 2021, a regional strategy for integrated disease surveillance was adopted by member states of the World Health Organization Eastern Mediterranean Region. But before then, member states including Somalia had made progress in integration of their disease surveillance systems. We report on the progress and experiences of implementing an integrated disease surveillance and response system in Somalia between 2016 and 2023.

**Methods:**

We reviewed 20 operational documents and identified key integrated disease surveillance and response system (IDSRS) actions/processes implemented between 2016 and 2023. We verified these through an anonymized online survey. The survey respondents also assessed Somalia’s IDSRS implementation progress using a standard IDS monitoring framework Finally, we interviewed 8 key informants to explore factors to which the current IDSRS implementation progress is attributed.

**Results:**

Between 2016 and 2023, 7 key IDSRS actions/processes were implemented including: establishment of high-level commitment; development of a 3-year operational plan; development of a coordination mechanism; configuring the District Health Information Software to support implementation among others. IDSRS implementation progress ranged from 15% for financing to 78% for tools. Reasons for the progress were summarized under 6 thematic areas; understanding frustrations with the current surveillance system; the opportunity occasioned by COVID-19; mainstreaming IDSRS in strategic documents; establishment of an oversight mechanism; staggering implementation of key activities over a reasonable length of time and being flexible about pre-determined timelines.

**Discussion:**

From 2016 to 2023, Somalia registered significant progress towards implementation of IDSRS. The 15 years of EWARN implementation in Somalia (since 2008) provided a strong foundation for IDSRS implementation. If implemented comprehensively, IDSRS will accelerate country progress toward establishment of IHR core capacities. Sustainable funding is the major challenge towards IDSRS implementation in Somalia. Government and its partners need to exploit feasible options for sustainable investment in integrated disease surveillance and response.

## 1. Introduction

Several countries in the Eastern Mediterranean Region of the World Health Organization (WHO) have disease control programs that have their own dedicated resources (for example, finances), structures (for example, information technology), tools, processes (for example, coordination, training and supportive supervision) and human resources. This approach is referred to as verticalization of disease surveillance. Vertical surveillance systems may be effective within their specific mandate, but from a systems perspective, verticalization causes avoidable duplication, redundancies and coordination challenges. Verticalization also creates surveillance gaps for diseases that do not have dedicated control programs (1). This adversely affects the efficiency and effectiveness of the surveillance system as a whole and argues in favor of a single multidisease surveillance system. The concept of a single multidisease surveillance system is based on the fact that surveillance systems for different disease control programs have many commonalities. Therefore, integrating these systems and using the same structures, personnel and processes to conduct surveillance for all priority diseases would lead to greater efficiency and effectiveness (2).

In 1999, the WHO Regional Office for Africa developed the integrated disease surveillance and response (IDSR) strategy (3). The IDSR strategy was adopted by all member states of the region and its implementation has resulted in improved efficiency and effectiveness, for example: improved overall quality of surveillance data, timely detection of outbreaks, improved consolidation of surveillance data leading to better adherence to reporting for the International Health Regulations (IHR) and data sharing requirements; and improved use of surveillance data for planning, monitoring and decision-making (4–12). However, momentum for integration of surveillance systems in member states of the WHO Eastern Mediterranean Region has been slow (13). Reasons for this include: the perception that surveillance system integration is for resource-constrained countries; the concern that surveillance system integration will dilute the quality of already established vertical surveillance systems; and the fear that surveillance system integration would lead to loss of jobs at the ministry of health (13).

Since 2008, the early warning alert and response network (EWARN) has been Somalia’s only multidisease surveillance system (14). Alongside EWARN, some disease control programs, such as polio and malaria, also have essentially vertical surveillance systems (15). In Somalia, despite context-specific challenges, EWARN has been effective in early detection and response to multiple disease outbreaks (14, 16). As specified in the 2020–2024 National Plan of Action for Health Security (NAPHS), Somalia made a decision to expand the coverage of EWARN to another 700 health facilities and, at the same time, to implement the integrated disease surveillance strategy to improve the overall performance of the national disease surveillance system (16). Surveillance system integration was revitalized in 2021 following a high-level multistakeholder workshop on surveillance system integration (17).

In 2021, using multiple lessons, the WHO Regional Office for the Eastern Mediterranean published a regional strategy for integrated disease surveillance (IDS) (1, 9, 12, 18). The goal of the IDS strategy is to guide member states on strengthening and integrating their national disease surveillance systems. The strategy identifies key actions that should be undertaken at the country level and are categorized under: (1) governance, (2) guidance, (3) surveillance tools, (4) appropriate resources, (5) data collection at the facility level, (6) data analysis and dissemination, (7) laboratory information management, (8) monitoring and evaluation, and (9) establishment of operational links between early warning and rapid response mechanisms (1). But before then, a few member states of the region had used guidance from elsewhere to make progress toward integration of their national disease surveillance systems, including Egypt, Jordan, Lebanon, Oman, Pakistan and Somalia (1).

In this study, we describe the progress in implementation of an integrated disease surveillance and response system (IDSRS) in Somalia for the duration 2016–2023. We also explore factors to which this progress is attributed. The findings are useful to member states of the region who wish to accelerate progress in integration of their surveillance systems.

## 2. Methods

### 2.1. Study design

We used a mixed methods design. First, we reviewed 20 relevant operational documents to identify key IDSRS implementation actions and/or processes undertaken or established between 2016 to 2023. Next, we conducted an online survey with 21 respondents to validate the identified actions and/or processes and to assess implementation progress using the integrated disease surveillance (IDS) monitoring and evaluation framework (1). Finally, we conducted 8 key informant interviews to explore the factors to which the expedient undertaking or establishment of these actions/processes is attributed (19).

### 2.2. Review of relevant operational documents

Twenty (20) relevant operational documents related to IDSRS planning and implementation for 2016–2023 were retrieved. The documents were reviewed to identify and extract actions and/or processes undertaken/established during the review duration. Accompanying text to fully describe the identified actions and/or processes was also extracted and summarized in narrative form. These were subsequently verified through an online anonymous survey.

### 2.3. Online survey

We retrieved the email addresses of 21 key persons who were engaged in the planning and implementation of integrated disease surveillance and response in Somalia and invited them to complete an anonymized online survey. Key persons who were engaged in IDSRS planning and implementation but are part of the authors were excluded from the survey. The respondents indicated the extent to which they agreed that each of the IDSRS implementation actions/processes identified from the reviewed documents was key. A given IDSRS implementation action or process was retained if more than 50% of the 21 respondents strongly agreed or agreed that it was key. The other actions/processes were dropped. The same online survey respondents also scored the extent of implementation (on a scale of 0, 25, 50, 75 and 100%) of each of the IDS actions as reflected in the IDS implementation monitoring and evaluation (M&E) framework. We calculated the mean progress score for every action (clustered according to pre-determined themes). We also calculated the mean progress score for all the actions under a specified theme to represent IDSRS implementation progress for that thematic area.

### 2.4. Key informant interviews

We asked online survey respondents to indicate whether they were interested in participating in a 10-min online key informant interview to further explore the factors influencing IDSRS implementation progress in Somalia. Interested respondents were scheduled and interviewed online. We used a standard guide to conduct the interviews, which were audio recorded. We continued with the interviews until saturation was achieved. We transcribed the audio recordings and analyzed the transcripts by inductive thematic analysis. We identified text segments relating to factors to which the current progress toward integrated disease surveillance and response strategy implementation is attributed. Text segments relating to the same reasons were given the same code. We summarized related codes under the same themes.

### 2.5. Reflexivity

Majority of the authors of this study are from WHO Somalia country office and the Somalia federal and state ministries of health. Majority of the authors of this study were also involved in the planning and implementation of IDSRS related activities or processes in Somalia. This facilitated interpretation of data for this study. None of the authors was a respondent or participant in this study. Also, none of the authors declared any interests that could have construed their objectivity during the design and implementation of this study.

### 2.6. Ethical considerations

Ethical approval for the study was obtained from the Somalia National Institute of Health (Ref NIH/IRB/02/Apr/2023). Permission to use program records was also obtained. Informed consent was sought from respondents and participants.

## 3. Results

### 3.1. Key IDSRS actions/processes implemented, 2016–2023

**Figure 1:**
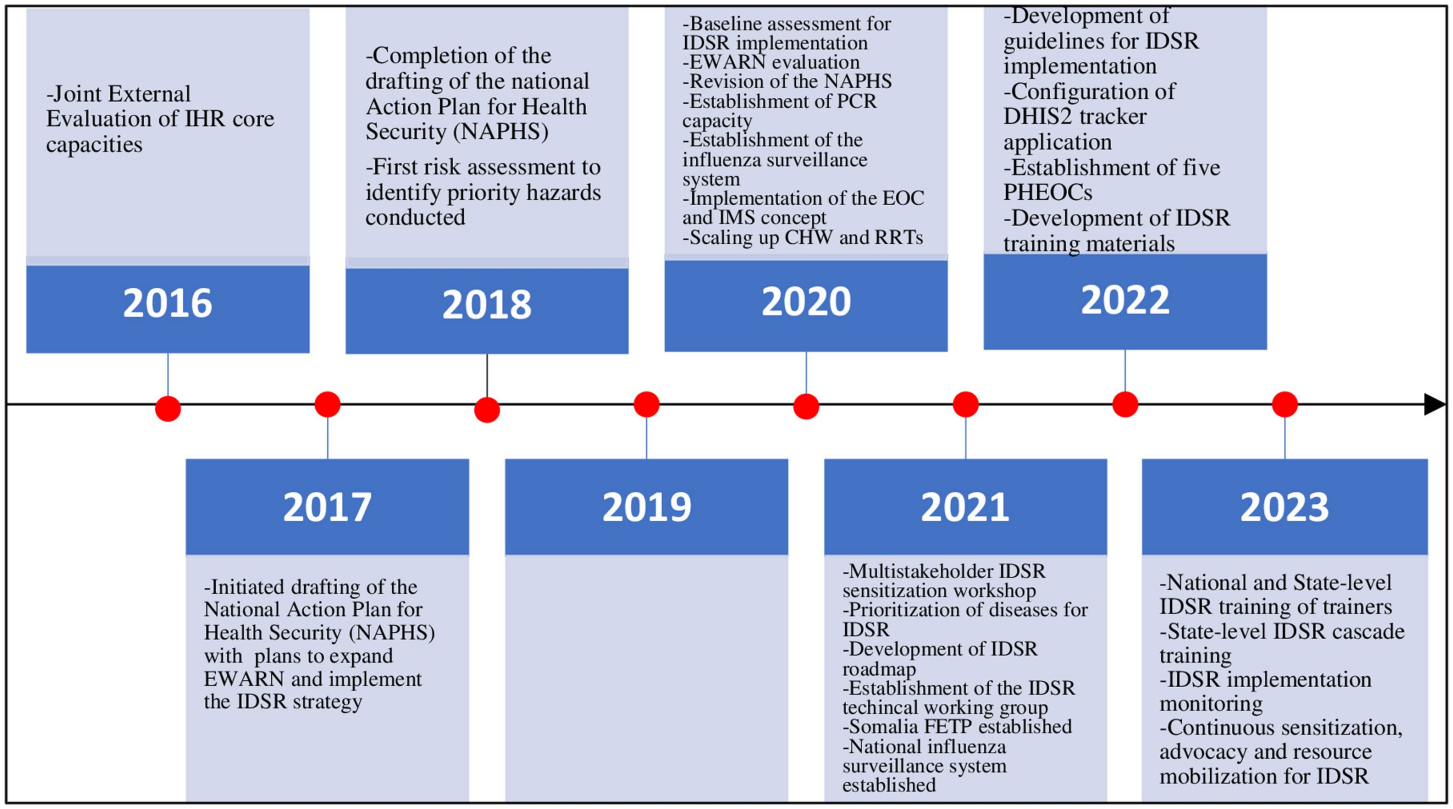
Key IDSRS actions/processes implemented, 2016–2023, Somalia. IHR, International Health Regulations; NAPHS, National Plan of Action for Health Security, EWARN, Early Warning Alert and Response, Network; IDSR, Integrated Disease Surveillance and Response; CO VID-19, Coronavirus Disease 2019, IMS, Incident Management System; EOC, Emergency Operations Centre; CHW, Community Health Worker; PCR, Polymerase Chain Reaction; FETP, Field Epidemiology Training Program; DHIS2, District Health Information Software 2; PHEOC, Public Health Emergency Operation Centre.

Twenty (20) documents were reviewed. These are summarized in Table 1. From these, a draft list of key IDSRS implementation actions/processes for the duration of 2016–2023 was developed. Next, a total of 21 respondents were surveyed online to validate the key actions and/or processes identified from reviewed documents. The characteristics of the survey respondents are summarized in Table 2. The respondents indicated the extent to which they agreed that each of the IDSRS implementation actions/processes identified from the reviewed documents was key. A given IDSRS implementation action or process was retained if more than 50% of the 21 respondents strongly agreed or agreed that it was key. The other actions/processes were dropped. A total of 7 IDSRS implementation actions/processes were retained. These are: (1) establishment of high-level commitment to improve the performance of the surveillance system using the integrated disease surveillance and response strategy; (2) establishment of stakeholder consensus on key features of the country’s integrated disease surveillance and response system; (3) development of a 3-year operational plan for implementation for integrated disease surveillance and response; (4) establishment of a mechanism for coordination of integrated disease surveillance and response system actors and actions; (5) development of technical guidelines for integrated disease surveillance and response; (6) configuration of the District Health Information Software 2 (DHIS2) tracker application to support reporting, alert management and other surveillance functions; and (7) development of training materials and conducting of training sessions as part of the broader plan for rolling out implementation of the developed technical guidelines for integrated disease surveillance and response to all levels of the health system in Somalia; Figure 1.

1. Commitment to implement the integrated disease surveillance and response strategy

**Table 1:** Documents reviewed to determine progress in implementation of integrated disease surveillance and response, 2016–2023, Somalia.

Operational document reviewed	Link to the reviewed operational document
Concept note on how IDSRS implementation can strengthen COVID-19 response in Somalia	https://drive.google.com/file/d/1kAI4kdy2mWp-VK0C-9-8CAZO__6s93z5/view?usp=sharing
Draft terms of reference for the IDSRStechnical working group	https://drive.google.com/file/d/1nlz3AOmvc3y5eqgV0DORY8e6cmjhq3tu/view?usp=sharing
Baseline assessment of existing surveillance systems in Somaliland	https://drive.google.com/file/d/1SerdvjCOBFnCOspeQYr94KXqIkSIxWMt/view?usp=sharing
Detailed 3-year operational plan for IDSRS implementation in Somalia	https://docs.google.com/document/d/1WLHVYht9IOTJpAT3oM7bKshXoHvTMFxZ/edit?usp=sharing&ouid=114290701937184242498&rtpof=true&sd=true
Detailed costing for the operational plan for IDSRS implementation in Somalia	https://docs.google.com/spreadsheets/d/1OMGYntfPgDhjQLGb9ARYb6f4cgEXEu4Z/edit?usp=sharing&ouid=114290701937184242498&rtpof=true&sd=true
Draft list of priority conditions for IDSRS in Somaliland	https://docs.google.com/document/d/1CNrD0iD8YRtmAyun0NEcDJeguBojRJkm/edit?usp=sharing&ouid=114290701937184242498&rtpof=true&sd=true.
Draft 3-year operational plan for IDSRS implementation in Somaliland	https://docs.google.com/document/d/1GULwkQDlUQZMYnS0G5Ci9qwHXfduH6fd/edit?usp=sharing&ouid=114290701937184242498&rtpof=true&sd=true
IDSRS strategy sensitization	https://docs.google.com/presentation/d/17GyxAN0wQIeL4CNWDQTgbxyhh3KRx_yL/edit?usp=sharing&ouid=114290701937184242498&rtpof=true&sd=true
Somaliland IDSRS and PHEOC workshop report	https://docs.google.com/document/d/11iFPDIEA5MBL_O-OjRrk-pdcvmKjreBW/edit?usp=share_link&ouid=114290701937184242498&rtpof=true&sd=true
Policy Briefs on IDSRS effectiveness	https://drive.google.com/file/d/1BwYpxJiAGPSLmfpM-XCjn0r54u5QpK2A/view?usp=sharing
IDSRS consultancy progress report 6 January 2021	https://drive.google.com/file/d/1FC3erbGy5XwgstAMjSXSJhiD8AlpEjdY/view?usp=sharing
IDSRS consultancy progress report 1 December 2022	https://docs.google.com/document/d/1dlJZHuScw823uyOq211LAAgkeMpBaq0V/edit?usp=share_link&ouid=114290701937184242498&rtpof=true&sd=true
IDSRS consultancy progress report 14 October 2022	https://docs.google.com/document/d/1dgU0pXdKb7oPsDp0g9PMcHYB8M1eSrEr/edit?usp=share_link&ouid=114290701937184242498&rtpof=true&sd=true
IDSRS consultancy progress report 15 June 2022	https://docs.google.com/document/d/18HJik0xU8ouUywdZeIkKgZI-VtcO7Hdf/edit?usp=share_link&ouid=114290701937184242498&rtpof=true&sd=true
IDSRS consultancy progress report 31 March 2022	https://docs.google.com/document/d/1uaU6oIS2Ar1jsEtcGp0_NaP-3GATJmma/edit?usp=share_link&ouid=114290701937184242498&rtpof=true&sd=true
IDSRS consultancy progress report 2 April 2021	https://docs.google.com/document/d/1vtQcYWp6AeKSgotdKkcZJWT-z9zlBIW5/edit?usp=share_link&ouid=114290701937184242498&rtpof=true&sd=true
DHIS2 tracker validation workshop report	https://docs.google.com/document/d/19mLF7hdw7e0uinpTV8tQaRcHnJRYybgX/edit?usp=share_link&ouid=114290701937184242498&rtpof=true&sd=true
IDSRS guidelines and operational plan validation: workshop report	https://docs.google.com/document/d/1t6n6wy9_aimv1uT0G97VlVp4-3lQL09n/edit?usp=share_link&ouid=114290701937184242498&rtpof=true&sd=true
IDSRS national and state level training of trainers: workshop report	https://docs.google.com/document/d/1rjkyfN1zWCC-grmic2m5xZgC3nggRqvh/edit?usp=share_link&ouid=114290701937184242498&rtpof=true&sd=true
IDSRS consultancy progress report 3 January 2023	https://docs.google.com/document/d/1qYNHgDFSgRdqmcbWs3sCdDCXLoqOOsVH/edit?usp=share_link&ouid=114290701937184242498&rtpof=true&sd=true

**Table 2:** Characteristics of online survey respondents and key informant participants.

Variable	Online survey respondents (*n* = 21)	Key informant participants (*n* = 8)
	No.	No.
Sex		
Male	15	5
Female	6	3
Age, in years		
< 30	4	1
31–40	14	5
> 40	3	2
Highest level of education		
Doctoral degree	3	0
Master’s degree	18	6
Bachelor’s degree	3	2
Current employer		
Federal Ministry of Health	8	4
State Ministry of Health	9	2
WHO	3	2
Other UN agency	0	0
Other	1	0
Area of specialization		
Disease surveillance	14	4
Data and information technology	3	2
Emergency coordination	2	2
Other	2	0
Length of time in the current position, in years		
< 1	1	0
1–5 years	13	6
> 5	7	2

In 2016, Somalia underwent a joint external evaluation of its IHR capacities to prevent, detect and respond to public health emergences. The country scored no or limited capacity in almost all the indicators related to surveillance (15). As a means to address the gaps identified, the Somalia national action plan for health security (NAPHS) was developed. The NAPHS identified and prioritized integrated disease surveillance and response as the overall strategy for addressing the gaps identified in the surveillance system (20). Other assessments that identified surveillance gaps were the surveillance system baseline assessment conducted in 2020–2021 and the early warning alert and response network (EWARN) system evaluation conducted in 2022 (14). Country commitment to IDSR strategy implementation was renewed in the wake of the COVID-19 pandemic which highlighted the urgent need to strengthen national disease surveillance systems using an integrated approach (21). The need to improve the performance of Somalia’s national disease surveillance system is highlighted in other strategic documents such as the current Somalia National Institute of Health operational plan and the current country cooperation strategy between WHO and Somalia (22, 23).

1. Consensus on the features of the country’s integrated disease surveillance and response system

**Figure 2:**
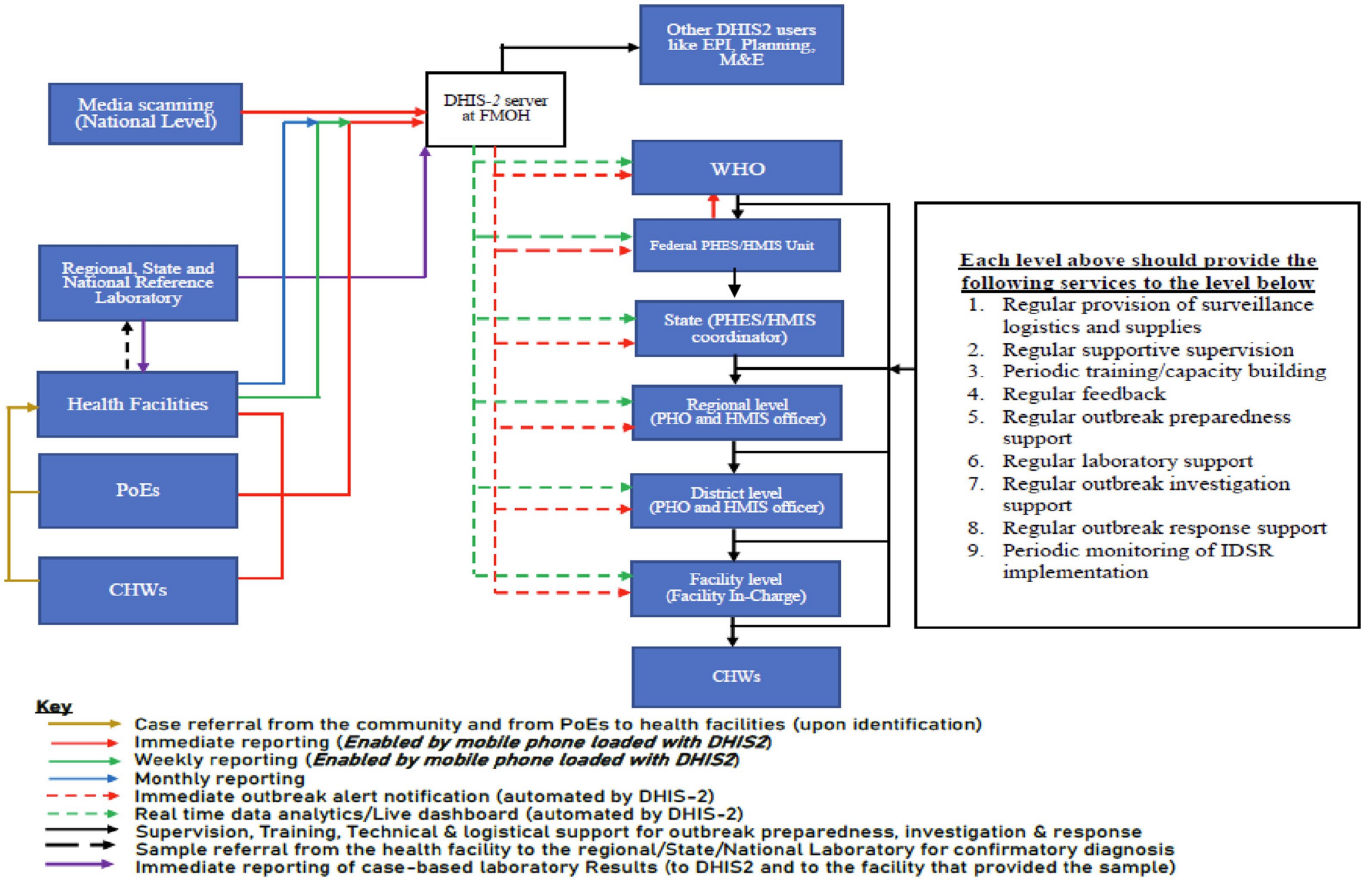
Revised flow of surveillance data and information in Somalia’s surveillance system.

In July 2021, a high-level multistakeholder workshop was held. The workshop was facilitated by WHO and was attended by more than 60 high-level officials from relevant ministries, departments and local and international agencies win Somalia including experts from Kenya, Nigeria, Uganda and the United Republic of Tanzania. The objectives of the workshop were to conduct sensitization and advocacy about integrated disease surveillance, generate consensus on key design features of the envisaged system and develop a draft roadmap for implementation of integrated disease surveillance in Somalia. An agreement was reached on key features of the desired disease surveillance system. These included: (1) revision of the list of priority conditions and their frequency of reporting; (2) all surveillance data to be integrated into the national health management information system (HMIS) database and to be hosted on a server maintained at the Federal Ministry of Health; (3) revision of the flow of surveillance data and information; and (4) selection of the DHIS2 tracker application as the information technology platform to support the implementation of integrated disease surveillance functions (17). The revised list of priority conditions for Somalia and their frequency of reporting are shown in Table 3. The revised flow of surveillance data and information in Somalia’s health system is outlined in Figure 2.

1. Development of a 3-year operational plan for implementation of integrated disease surveillance

**Table 3:** Revised list of priority conditions for IDSRS and their frequency of reporting, Somalia.

No.	Priority disease/condition	Reporting frequency		
1	Acute flaccid paralysis	Immediate	Weekly	
2	Acute haemorrhagic fever syndrome*	Immediate	Weekly	
3	Acute jaundice syndrome	Immediate	Weekly	
4	Acute watery diarrhea/cholera	Immediate	Weekly	
5	Adverse events following immunization	Immediate	Weekly	
6	Anthrax	Immediate	Weekly	
7	Bloody diarrhea	Immediate	Weekly	
8	Brucellosis	Immediate	Weekly	
9	Cluster of unexpected deaths	Immediate	Weekly	
10	Cluster of unexpected illness	Immediate	Weekly	
11	COVID-19	Immediate	Weekly	
12	Human rabies	Immediate	Weekly	
13	Cluster of influenza-like illness	Immediate	Weekly	
14	Measles	Immediate	Weekly	
15	Meningococcal meningitis	Immediate	Weekly	
16	Poliomyelitis (laboratory confirmed)	Immediate	Weekly	
17	Radiological/chemical event/foodborne illness	Immediate		
18	Cluster of severe acute respiratory illness	Immediate	Weekly	
19	Trypanosomiasis	Immediate	Weekly	
20	Typhoid fever	Immediate	Weekly	
21	Yellow fever	Immediate	Weekly	
22	Leprosy	Immediate	Weekly	
23	Lymphatic filariasis		Weekly	
24	Maternal death		Weekly	
25	Neonatal death		Weekly	
26	Neonatal tetanus		Weekly	
27	Perinatal death		Weekly	
28	Acute and chronic hepatitis			Monthly
29	Confirmed malaria cases	Immediate	Weekly	
30	Diabetes			Monthly
31	HIV/AIDS (new cases)			Monthly
32	Hypertension			Monthly
33	Injuries/trauma			Monthly
34	Intestinal worms			Monthly
35	Malnutrition in children younger than 5 years			Monthly
36	Mental disorders			Monthly
37	Multidrug resistant tuberculosis			Monthly
38	Schistosomiasis			Monthly
39	Severe pneumonia in children younger than 5 years			Monthly
40	Sexually transmitted infections			Monthly
41	Trachoma			Monthly
42	Tuberculosis (new cases)			Monthly

^*^Ebola virus disease, Marburg virus disease, Rift Valley fever, Dengue and Chikungunya.

**Figure 3:**
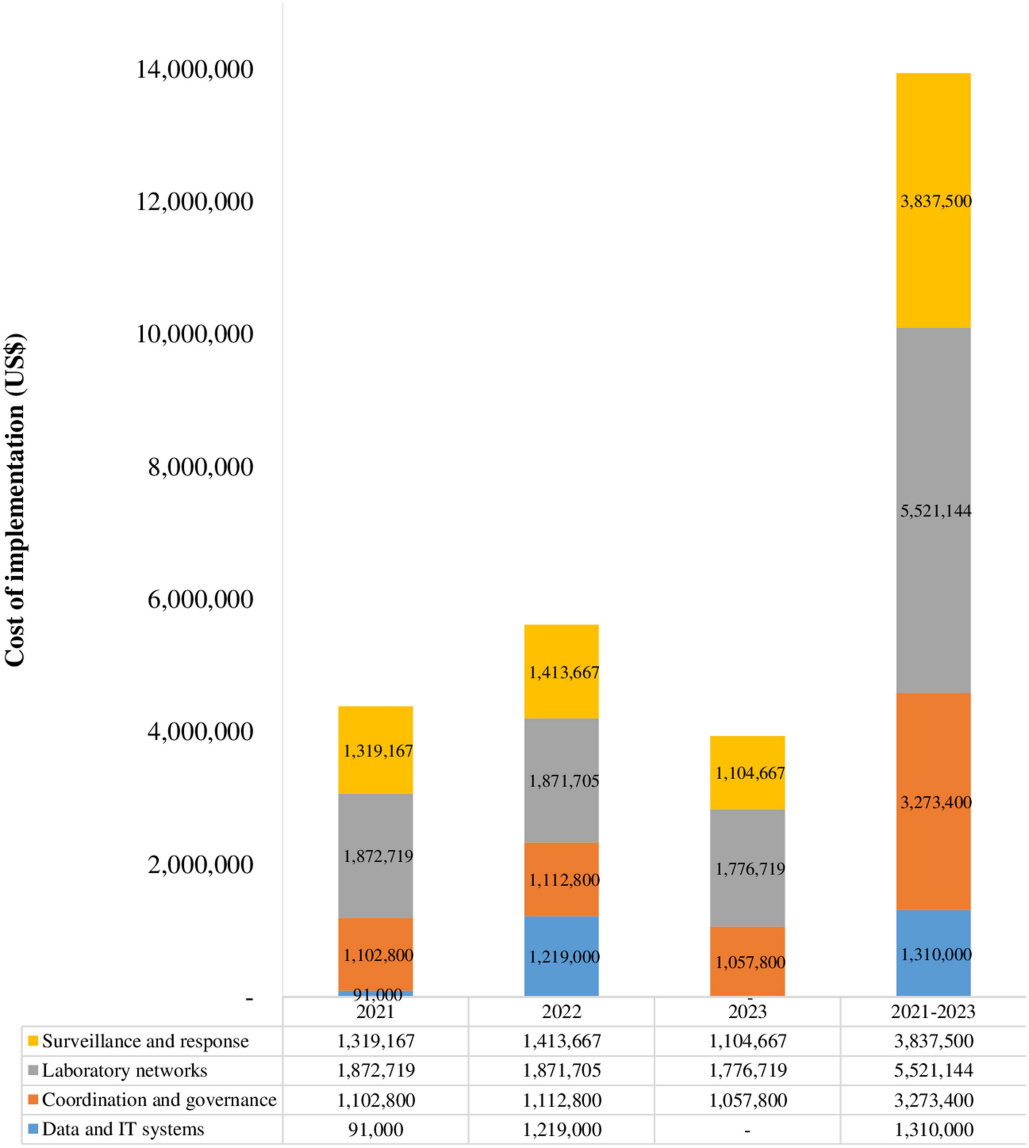
Estimated cost of implementation of integrated disease surveillance, 2021-2023.

One of the outputs from the July 2021 high-level advocacy and sensitization workshop on implementation of integrated disease surveillance in Somalia was the development of a draft operational plan for implementation integrated disease surveillance in Somalia. The plan was finalized by the Federal Ministry of Health with technical support from the WHO country office. The plan covers 2021–2023 and includes key activities to be implemented in the 3 years. The activities are clustered under four thematic areas: (1) coordination; (2) data and information technology systems; (3) laboratory networks; and (4) surveillance and response. For every activity, the implementation timeline and the unit with overall responsibility for its implementation are specified. Through multiple consultations, the costed plan was reviewed, validated and endorsed by Federal Ministry of Health. The cost of implementing the 3-year plan is estimated at US$ 14000000. Breakdown of the cost by year and thematic area is given in Figure 3.

1. Early establishment of a technical working group on integrated disease surveillance

The FMOH with support from WHO developed terms of references (ToRs) for a multisectoral technical working group (TWG) on integrated disease surveillance and response. The ToRs were reviewed during the July 2021 high level workshop and later refined and endorsed. The TWG was conceived as one of the sub-committees of the IHR committee. Its composition includes technical representatives from government and non-government ministries, departments and agencies supporting disease surveillance and response in Somalia. Among others, its functions include; provision of technical oversight in the development of the operational plan and technical guidelines for integration of surveillance systems; leading on advocacy and sensitization on integrated disease surveillance; mobilization of resources for implementation of the operational plans and guidelines for surveillance system integration; and overseeing the implementation of surveillance related capacity building interventions. The TWG conducts its business through bi-weekly meetings chaired by NIH co-chaired by WHO Somalia country office. The TWG was very instrumental in first tracking the development, completion and endorsement of the operational plan and technical guidelines for integrated disease surveillance in Somalia.

1. Development of technical guidelines for integrated disease surveillance and response

A team with experience in surveillance and guideline development was nominated to lead the development of the Somalia national technical guidelines for implementation of integrated disease surveillance. The team followed the ADAPTE framework for guidelines development (24, 25) and reported their progress in the biweekly technical working group meetings on integrated disease surveillance. Instead of developing new guidelines from scratch, the team selected the third edition of the generic guidelines on integrated disease surveillance and response of WHO African Region as the source guidelines (26). The team retrieved available material related to communicable surveillance and response (CSR) and EWARN implementation and used these to adjust the source guidelines to the Somali context. The draft guidelines were reviewed both internally and externally and in a validation workshop. After each review, necessary adjustments in the guidelines’ content were made. In September 2022, the guidelines were endorsed by the federal minister of health as the primary source of all the required tools for conducting surveillance functions at any level of the health system in Somalia. To make the Somalia guidelines more operational, the overall size was reduced from more than 600 pages (source guidelines) to fewer than 100 pages, and for each surveillance function, an easy–to-implement standard operating procedure was developed.

1. Customization of the DHIS2 tracker application to automate surveillance system integration

District health information software version 2 (DHIS2) is the Somalia’s preferred platform for enabling transformation from manual standalone to automated and integrated disease surveillance (27). Since 2020, health information strengthening project/university of Oslo (HISP/UiO) has supported Somalia to customize the DHIS2 tracker application (an extension of DHIS2) according to the new guidelines for surveillance system integration in Somalia. DHIS2 capture, which is the mobile component of the DHIS2 tracker application has been customized to support both the immediate (case-based) and weekly (aggregate) reporting components as envisaged in the new country integrated disease surveillance guidelines. Using the DHIS2 tracker capture, health facility workers and community volunteers will be able to report alerts of priority conditions in a standardized manner direct into DHIS2 without the necessity to complete manual forms and send them to the district. The customized DHIS2 tracker will also automate aggregation, analysis and visualization (through user customized dashboards) of surveillance data and its integration into the national health information system database. Multiple stakeholder engagements were conducted to review and validate the customized DHIS2 tracker application. The roll-out of the customized DHIS2 tracker application was integrated into the broader plans for rolling-out the new integrated disease surveillance guidelines.

1. Development of training materials and training sessions

After endorsement of the integrated disease surveillance guidelines, the adaptation team developed training materials based on the guidelines and the WHO African Region’s training course integrated disease surveillance (28). The training materials included PowerPoint presentations, and a facilitators and participants guide. They were developed in modular format, with each module corresponding to a particular surveillance function. The modules are: introduction; module 1 detection and recording; module 2 reporting; module 3 analysis; module 4 outbreak investigation; module 5 preparedness; module 6 response; module 7 risk communication and community engagement; module 8 monitoring; module 9 supportive supervision; module 10 evaluation; and a hands-on session on how to use the DHIS2 tracker application to support reporting, alert management and other functions. The materials put emphasis on the skills required to implement each of the surveillance functions. At the end of each module, 1–2 skills building exercises are included. The training materials were also thoroughly assessed by many internal and external reviewers before their use.

**Figure 4:**
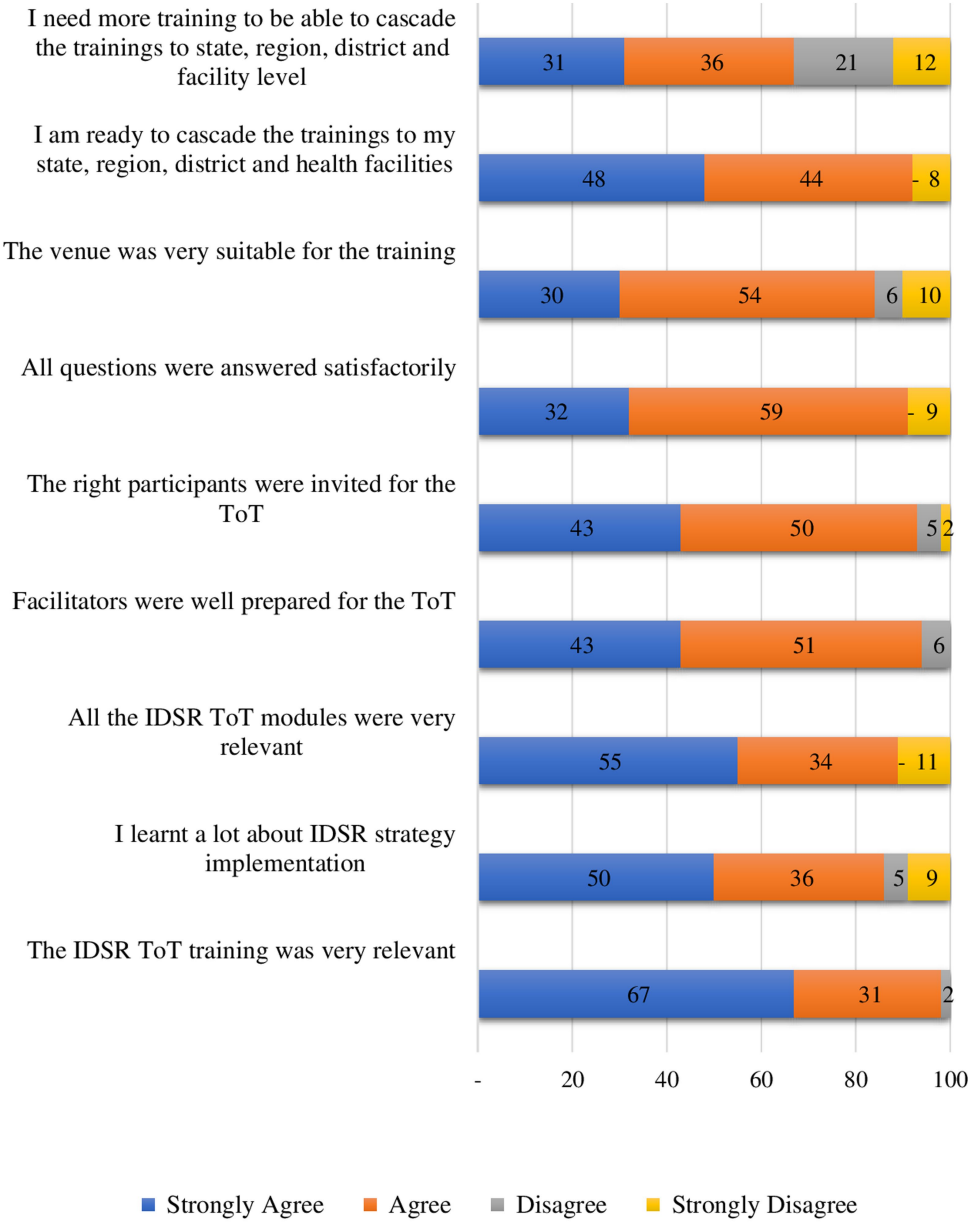
Participant evaluation of the training-of-trainers course on implementation of integrated disease surveillance, Somalia, 2023.

In February 2023, a national and state-level training-of-trainers course on implementation of integrated disease surveillance was conducted. The objective was to equip federal and state teams with the knowledge and skills required to conduct integrated disease surveillance cascade training and to lead and supervise integrated disease surveillance implementation. The training was for 5 days and was facilitated by staff of WHO, Federal Ministry of Health, National Institute of Health and Health Information Strengthening Project (HISP) Tanzania. A total of 53 participants were trained and on the last day, participants worked in groups to develop draft plans for cascading the training in their states. Figure 4 shows that more than 90% of the participants felt that they were ready to undertake the cascade training in their states.

### 3.2. Implementation progress according to the IDS monitoring and evaluation framework

**Figure 5:**
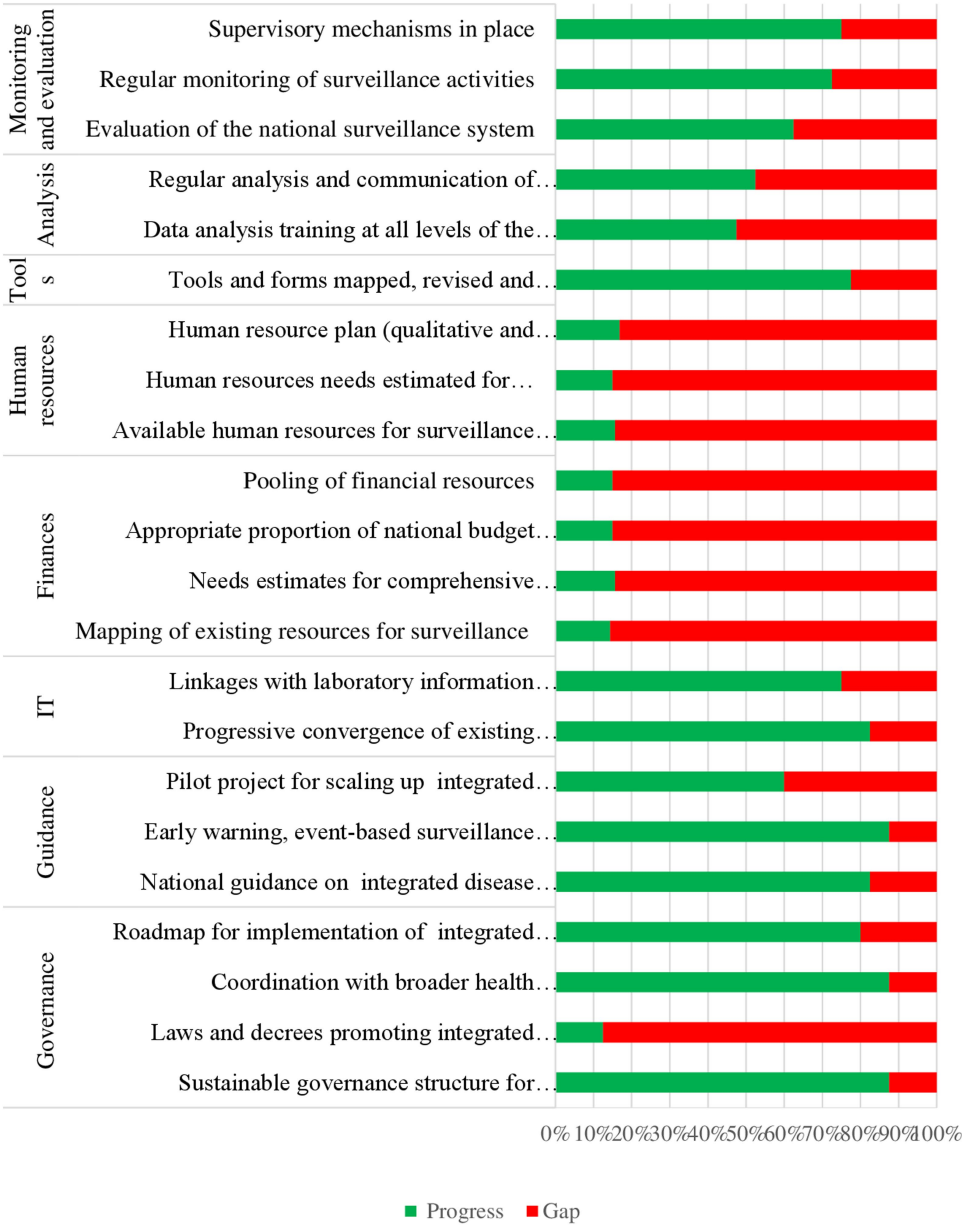
IDSR strategy implementation progress, 2016-2023, Somalia.

The online survey respondents also scored the extent of implementation (on a scale of 0, 25, 50, 75 and 100%) of each of the IDS actions as reflected in the IDS implementation monitoring and evaluation (M&E) framework (1). We calculated the mean score for every action (clustered according to pre-determined themes). We also calculated the mean score for all the actions under a specified theme to represent IDSRS implementation progress for that thematic area. Basing on the results of this analysis, IDSRS implementation progress in Somalia was rated at: 67% for governance, 77% for guidance, 79% for information technology, 15% for finance, 16% for human resources, 78% for tools, 50% for data analysis, and 70% monitoring and evaluation. This is summarized in Figure 5.

### 3.3. Factors to which the current IDSRS implementation progress is attributed

Eight key informants were interviewed on factors to which the current IDSRS implementation progress was attributed. Four of these worked in the Federal Ministry of Health and four worked in disease surveillance and response. The characteristics of key informants are summarized in Table 2. A thematic analysis of transcripts from 8 key informant interviews generated multiple factors to which the current IDSRS implementation progress in Somalia is attributed. These factors were summarized under six (6) themes. These are: (1) understanding frustrations with the current surveillance system; (2) utilizing opportunities resulting from the COVID-19 pandemic; (3) mainstreaming surveillance system integration in strategic documents; (4) establishing a mechanism for oversight of integration efforts; (5) applying a holistic/whole of society/systems thinking approach toward integration of the surveillance system; and (6) staggering implementation over a reasonable length of time and being flexible about pre-determined timelines.

1. Understanding of the frustrations with the current surveillance system

Initially, planners, managers and users of the disease surveillance system in Somalia perceived the challenges with the current surveillance system differently. Several were frustrated that some diseases were not included in the current system. Some were concerned that the system’s geographical coverage was limited. Others were dissatisfied that the system was not providing feedback to the lower levels. Many stakeholders wanted to have more control over the surveillance system. During the high-level multistakeholder workshop in 2021, consensus was reached on many design issues, including the list of priority conditions for surveillance, the information technology platform, hosting of surveillance data and right of access to the data. Agreement on the overall goal of integration of the surveillance system has been central to shared effort and acceleration of integration.

We had our own individual preferences, but we cannot do that now because we have a joint roadmap. Everyone knows and agreed what we should do and when we should do it. This is very important for better coordination and joint tracking of progress (Key informant 2).

The concept of system integration was also understood differently. Some perceived that the country had embarked on developing a new surveillance system with a new personnel structure. Others reduced integration to an information technology platform for reporting of diseases and nothing beyond that. In fact, representations of a surveillance system often reduce it to the flow of information. In the case of Somalia, a model showing the inputs (such as technical guidelines) feeding into support functions to feed into core functions to realize the goals of system integration was a key tool to create an understanding of what integration of disease surveillance systems was and what it was not.

…it clearly showed (the model for integrated surveillance) why our current system was collapsing. For example, we were not doing supportive supervision because we did not have enough resources. Gradually, facilities lost interest and stopped reporting (Key informant 4).

1. COVID-19 pandemic and momentum for surveillance system strengthening

The momentum for surveillance system integration coincided with the COVID-19 pandemic. It was during the COVID-19 response that mechanisms such as emergency operation centers and the incident management systems were put in place in Somalia. The fact that these systems were effective for COVID-19 control and because they are also core components of surveillance system integration provided support for the scaling up of these and other related systems.

During the early days of COVID-19, we established an incident management team composed of experts from different units. With support from WHO, we quickly deployed community health workers in communities that are far from health facilities and rapid response teams at the district level. So, we now know that these concepts work (Key informant 5).

1. Mainstreaming surveillance system integration in strategic documents

The importance of strengthening Somalia’s disease surveillance system through integration is made clear in multiple health strategic documents. Among other documents, these include the current health sector strategic plan, the NAPHS, the strategic plan of the Somalia National Institute of Health and the current Country Cooperation Strategy between WHO and Somalia. These documents also identify the same priorities for integration of the surveillance system. Somalia also developed and costed a 3-year operational plan for implementation of priority activities for surveillance system integration. These activities fell under four thematic areas: (1) governance and coordination; (2) data and information technology systems; (3) laboratory and networks; and (4) surveillance and response. The plan indicated the approximate cost of implementing each activity, the unit responsible for leading the activity and timelines for its completion. The operational plan also broadened stakeholders’ understanding that surveillance system strengthening is a sector-wide reform. The operational plan was the main document used to advocate for and mobilize resources for surveillance system integration and to monitor progress in surveillance system integration.

1. Establishing a mechanism for coordinating integration efforts

Improved coordination of actions and actors across multiple ministries, departments and agencies supporting surveillance is an important part of surveillance system integration. Early on, with technical support from WHO, terms of reference for the establishment and operationalization of a coordination mechanism were developed. A technical working group was established co-chaired by National Institute of Health and the WHO country office. The working group has provided strategic oversight in advocating for and monitoring implementation of the integrated disease system. While initially the group was very active, over time, meetings became less frequent and its composition did not expand to include representatives from other critical units/departments, such as human resources and finance and other relevant ministries and non-human health sectors.

……I think we do not yet have the mandate or the other ministries do not perceive that the ministry of health can coordinate them. So, even when we invite them and they do not attend, there is nothing much we can do (Key informant 6)

The perception that implementation of the surveillance system integration operational plan is slow has also negatively affected the functioning of the technical working group.

…. for example, on one day, you might be required to attend say five meetings. So, if you do not get someone to represent you in one, you postpone it or open it and leave them discussing as you go to attend another one (Key informant 6).

As has been noted with multisectoral collaboration efforts elsewhere, these challenges are not unexpected. What is more important is to have the leadership and management skills to unblock such stagnations and maintain momentum when stakeholders begin to disengage because of perceived slow progress or for any other reason. With technical support from the WHO country office, the Federal Ministry of Health and National Institute for Health are trying to maintain the functioning of the technical working group and intensify stakeholder sensitization and advocacy for surveillance system integration.

1. Applying a holistic/whole of society/systems thinking approach toward surveillance systems integration efforts

The surveillance system does not exist in a vacuum. It is part of the broader health system. The surveillance system will be as strong as or as weak as the health system within which it functions. The Federal Ministry of Health and National Institute for Health are therefore working to mobilize support to address the broader determinants of performance of the country’s health system. Notably, in collaboration with WHO, Intergovernmental Authority on Development and Centers for Disease Control and Prevention, they are implementing the Frontline Field Epidemiology Training Program. This program aims to build human resources capacity for frontline surveillance and response. The WHO country office is also supporting the strengthening of national laboratory systems and the United Nations Children’s Fund (UNICEF) is supporting the strengthening of the country’s health information management system. Strategies to address bottle-necks in the broader determinants of surveillance system performance will help accelerate implementation of integrated disease surveillance system.

1. Staggering implementation of implementation activities

During the development of the operational plan for surveillance system integration, many actions were suggested to strengthen the system to be implemented within 3 years. The planning team acknowledged that multiple improvements were required but that not all required actions could be implemented in current system at the same time or in a short duration. Therefore, it was agreed that only the essential activities would be implemented during the 3 years of the operational plan.

Yes, configuring the DHIS2 tracker application took more time than we had planned. But the team was making good progress and they were developing a good system. We gave them time (Key informant 1).

## 4. Discussion, study limitations, conclusion and next steps for Somalia

### 4.1. Discussion

We have described the progress in implementation of an integrated disease surveillance and response system (IDSRS) for the time interval 2016–2023 in Somalia. Quantitatively, country progress depending on which thematic area ranges between 15 and 79%. Specifically, the following have been achieved; establishment of high-level commitment to implement an integrated disease surveillance and response system; establishment of stakeholder consensus on key features of the country’s integrated disease surveillance and response system; development of a 3-year operational plan for implementation for integrated disease surveillance and response; establishment of a mechanism for coordination of integrated disease surveillance and response system actors and actions; development of technical guidelines for integrated disease surveillance and response; configuration of the District Health Information Software 2 (DHIS2) tracker application to support reporting, alert management and other surveillance functions; and development of training materials and conducting of training as part of the broader plan for rolling out the developed technical guidelines for integrated disease surveillance and response to all levels of the health system in Somalia.

The results on IDSRS implementation progress for the duration 2016–2023 in Somalia are mixed; reflecting good progress in some areas and limited progress in others. For example, by the time of this study, the customized DHIS2 tracker capture application had not yet been deployed onto the mobile phones of health workers. Equally, apart from a national IDSRS roll-out training of trainers (ToT), cascade trainings (including how to use the new DHIS2 tracker application) were planned but had not been conducted. Steps of IDSRS implementation are systematic. Subsequent steps are dependent/contingent upon completion of previous steps. As an example, even if training materials are ready, trainings could not be conducted until the configuration of the DHIS2 tracker capture application was complete. Additionally, the need for meaningful engagement of multiple stakeholders meant that IDSRS related workshops would only be held if all or majority of relevant stakeholder representatives were available. The dependence of subsequent steps on previous steps; the requirement to conduct workshops when all relevant stakeholders are available often caused implementation delays. Elsewhere, taking time/being flexible with previously set timelines has been identified as a requirement for building critical collaborative relationships, co-construction of reality, and building a critical mass of understanding which are key ingredients for successful implementation of integrated disease surveillance and response and other related collaborative interventions (29).

A few health facilities (307 out of the expected about 900 across the whole country) were however already submitting their weekly aggregate surveillance data not directly into DHIS2 but to the district public health officer who would then submit the manually received data into DHIS2 using the desktop version of DHIS2 tracker capture. The federal level was using this data to generate and widely disseminate epidemiological bulletins on a weekly basis. By the 7^th^ epidemiological week of 2023, 234 out of these 307 health facilities were submitting their weekly aggregate surveillance data, representing a completeness of 76% (30). By the same time, there was however no immediate case-based data because the immediate reporting component of the DHIS2 tracker capture application is not desktop enabled. Cascade trainings (which also include deploying the DHIS2 tracker capture application onto the mobile phones of health workers) were planned to begin in April 2023 after which both immediate case-based and more weekly aggregate surveillance summary reports were expected to be submitted automatically, direct from the health facilities into DHIS2 using the deployed android phone enabled DHIS2 tracker capture application.

Somalia is/did not implement IDSRS from scratch. The 15 years of EWARN implementation in Somalia (since 2008) provided a strong foundation for IDSRS implementation. During this time, the number of health facilities participating in surveillance has been expanded to 689 health facilities in 64% of all the districts in Somalia. Health workers have been trained repeatedly on how to detect and report cases of priority conditions, how to verify and investigate alerts of epidemic prone conditions including collection, packaging, transportation and testing of appropriate laboratory specimen, outbreak investigation, surveillance data analysis among other surveillance functions (14). As the strategy for transitioning from EWARN in Somalia, IDSRS implementation will seek to maintain and build on the 15 years surveillance system gains which are attributable to EWARN implementation in Somalia.

Findings of the 2016 joint external evaluation of international health regulation (IHR, 2005) capacities revealed that globally, Somalia has the least developed health security architecture (31). This was further illuminated when huge investments in terms of; human resources at both facility and community level, Information Technology (IT); and laboratory capacity (in terms of equipment, reagents and supplies) had to urgently be mobilized and deployed to plug the huge surveillance system gaps to support the country’s response to the COVID-19 pandemic. In the country’s national action plan for health security (NAPHS) and other related strategic documents, IDSRS is identified as the major driver for accelerating progress toward establishment of international health regulations (IHR, 2005) capacities to prevent, detect and respond to outbreaks and other health security threats (23, 27, 32–34). This is the same approach that member states in the African region of WHO took (10).

Implementation of IDSRS in Somalia will address multiple gaps in the country’s core capacities for IHR. IDSRS will also align the actions and resources of multiple government and non-government partners to jointly defined surveillance priorities, integrate surveillance functions and avoid resource duplications and redundancies, consolidate data on multiple diseases, levels and systems into a single dataset and integrate it into the national health information system database resulting into a robust system for detection and response to outbreaks and other acute public health threats (16).

However, IDSRS implementation comes with a number of challenges. For example, established vertical surveillance systems are often hesitant to integrate into a single multidisease surveillance system. The continued sensitization, advocacy, negotiation and coordination with units with vertical surveillance systems and their partners will be key to successful implementation of an integrated disease surveillance and response system in Somalia. Somalia being fragile and in a humanitarian emergency setting, the other huge challenge is sustainable investment in IDSRS implementation. This is especially true in terms of human resource capacity (numbers, inadequate training) and availability of sufficient finances to support IDSRS implementation (12). To address the challenge of critical shortage of human resources for IDSRS implementation, and to build a strong cadre of disease detectives in line with IHR requirements, with technical support from WHO Somalia country office, the FMOH and NIH are implementing the Somalia frontline field epidemiology training program (SOM-FETP-Frontline) (35, 36). The sustainability of this and other system-wide interventions will require continued mobilization of resources and alignment of donor support to IDSRS implementation priorities and the other priority health security strengthening interventions as outlined in the national action plan for health security (NAPHS) and other related national strategic plans. One of such opportunities is the World Banks Pandemic call for proposals for funding of up to USD 300000000 available to support developing countries to better prepare for and respond to future pandemics (37). Accessing these and other funds like from Global fund and aligning them to Somalia’s national plan of action for health security will go a long way toward building sustainable capacity to prevent, quickly detect and expediently respond to outbreaks and other acute public health events in Somalia.

### 4.2. Study limitations

Majority of reviewed documents were commissioned by WHO Somalia country office. The content in these documents could have been biased toward what is acceptable by both WHO and the federal and state ministries of health. To reduce this potential bias, data extracted from these documents was triangulated through an online survey. But also, majority of the online survey respondents were from WHO and federal and state ministries of health. To reduce the likelihood of social desirability bias, the need for objective responses to the survey questions was clearly described in the consent form. Also, the survey was anonymized.

### 4.3. Conclusions and next steps for Somalia

From 2016 to 2023, Somalia registered significant progress toward implementation of an integrated disease surveillance and response system. IDSRS if implemented comprehensively will scale up the 15-year gains of EWARN and accelerate country progress toward establishment of IHR core capacities and a robust system to prevent, detect and respond to outbreaks and other acute public health threats. The use of IDSRS implementation to scale-up EWARN gains will include conducting surveillance and response in more health facilities (more than the 689 health facilities that were participating in EWARN), conducting surveillance and response on more conditions (14 conditions for EWARN versus 29 conditions for IDSRS), collecting more data on each condition reported through IDSRS, conducting more analysis on submitted IDSRS data, providing access to surveillance data dashboard and wider dissemination of weekly surveillance data analytics to more stakeholders at multiple levels of the health system than it was possible during EWARN implementation. This means that compared to EWARN, IDSRS will improve Somalia’s capacity to detect and respond to outbreaks caused by more priority conditions. The government and its partners need to exploit feasible options for sustainable investment in system -wide interventions required to strengthen IDSRS implementation in Somalia. The next steps toward include phased roll-out of IDSRS to all levels of the health systems and continued mobilization of equipment, logistics, supplies and other resources required for successful roll-out and sustainable IDSRS implementation.

## Data availability statement

The raw data supporting the conclusions of this article will be made available by the authors, without undue reservation.

## Ethics statement

The studies involving humans were approved by Somalia National Institute of Health (Ref NIH/IRB/02/Apr/2023). The studies were conducted in accordance with the local legislation and institutional requirements. The participants provided their written informed consent to participate in this study.

## Author contributions

SS, MK, AA, and ML conceived the study. SS, MBM, MAM, and AD supported the ethical approval. SS, MK, AA, and ML contributed to data acquisition and data analysis. SS drafted the manuscript and revised the manuscript. MK, AA, ML, AAA, KM, AO, AhMA, MA, AbMA, AM, HS, NH, AH, AI, AYM, IN, MBM, MAM, FN, HM, MD, AD, and MRM reviewed the manuscript. All authors contributed to the article and approved the submitted version.

## Conflict of interest

The authors declare that the research was conducted in the absence of any commercial or financial relationships that could be construed as a potential conflict of interest.

## Publisher’s note

All claims expressed in this article are solely those of the authors and do not necessarily represent those of their affiliated organizations, or those of the publisher, the editors and the reviewers. Any product that may be evaluated in this article, or claim that may be made by its manufacturer, is not guaranteed or endorsed by the publisher.
